# Protective effect of Garcinia against renal oxidative stress and biomarkers induced by high fat and sucrose diet

**DOI:** 10.1186/1476-511X-10-6

**Published:** 2011-01-14

**Authors:** Kamal A Amin, Hamdy H Kamel, Mohamed A Abd Eltawab

**Affiliations:** 1 Biochemistry Department, Faculty of Vete. Medicine, Beni-Suef University, Beni-Suef, Egypt; 2 Clinical pathology Department, Faculty of Vete. Medicine, Beni-Suef University, Beni-Suef, Egypt

## Abstract

**Background:**

Obesity became major health problem in the world, the objective of this work was to examine the effect of high sucrose and high fat diet to induce obesity on antioxidant defense system, biochemical changes in blood and tissue of control, non treated and treated groups by administration of Garcinia cambogia, and explore the mechanisms that link obesity with altered renal function

**Methods:**

Rats were fed a standard control diet for 12 week (wk) or a diet containing 65% high sucrose (HSD) or 35% fat (HFD) for 8 wk and then HFD group divided into two groups for the following 4 wks. One group was given *Garcinia*+HFD, the second only high fat, Also the HSD divided into two groups, 1^st ^HSD+*Garcinia *and 2^nd ^HSD. Blood and renal, mesenteric, Perirenal and epididymal adipose tissues were collected for biochemical assays.

**Results:**

HFD and HSD groups of rats showed a significant increase in feed intake, Body weight (BW) and body mass index (BMI). Also there were significant increases in weights of mesenteric, Perirenal and epididymal adipose tissues in HFD and HSD groups.

HFD and HSD affect the kidney by increasing serum urea and creatinine levels and decreased level of nitric oxide (NO) and increased blood glucose, low density lipoproteins (LDL), triacylglycerol (TG), total cholesterol (TC) and malondialdehyde (MDA). Glucose 6-phosphate dehydrogenase (G6PD) activities were significantly decreased in HFD while there were significant increases in HSD and HSD+G groups p ≤ 0.05 compared with control. Moreover, renal catalase activities and MDA levels were significantly increased while NO level was lowered. These changes improved by *Garcinia *that decreased the oxidative stress biomarkers and increased NO level.

There were significant positive correlations among BMI, kidney functions (Creatinine and urea), TG and Oxidative markers (renal MDA and catalase).

**Conclusions:**

Rats fed a diet with HFD or HSD showed, hypertriglyceridemia, increased LDL production, increased oxidative stress and renal alteration. Moreover, suggesting association between lipid peroxidation, obesity and nephropathy, while *Garcinia *ameliorated the damaging effects of the HFD or HSD and decreased feed intake, MDA level and decreased oxidative stress in renal tissues.

## Introduction

Overweight and obesity affected 1.6% of 2-6 year olds, 4.9% of 6-10 year olds, 14.7% of 10-14 year olds, and 13.4% of 14-18-year-old children. The prevalence overweight and obesity were 45.3 and 20% respectively in Egyptian urban men, while they were 28.1% and 6.0% respectively in rural men [1].

Over the past 15 years, there has been an increase in the scientific interest in the impact of high fat and sucrose diet on health. Several experimental studies have been published on the cardiovascular (CV) and hepatic relation to obesity. There was a shortage of data and research about the effect of obesity on renal pathophysiology and biochemical relationship which deserves further investigation.

The pathogenesis of diabetic nephropathy remains far from obvious, partly due to the lack of a suitable animal model that mimics human renal disease in type 2 diabetes. Therefore our aim was to develop rodent model of obesity by HFD and HSD and to demonstrate the biochemical changes and mechanisms that link obesity with altered renal function and its treatment with *Garcinia cambogia*. Also this study evaluates the contribution of the serum and/or renal NO, oxidative stress and renal function to the renoprotection conferred by the *Garcinia *in rats.

## Materials and methods

This study was approved by the Committee of Scientific Ethics at Beni Suef University and according to its guidelines.

### Material

#### 1- Diet

three types of diets have been used, control rat chow diet, special HFD (35%) and special HSD (65%) for induction of obesity in rats.

**a- Normal rat chow diet: **It was formed according to Kim et al. [2]. The standard normal rat chow consists of concentrate (350 gm), corn (600 gm), calcium carbonate, dicalcium phosphate, sodium chloride magnesium oxide and vitamins (50 gm).

Standard normal rat diet composed of 65% CHO (60% starch+5% sucrose), Fat 5%, crude protein 20%, vitamins and minerals 5%, fibers 5%, metabolic energy of this diet is 2813 Kcal/Kg with 8% from fat

**b- The high fat diet**, composed of 300 gm concentrates, 350 corn, 300 gm beef tallow, 50 gm vitamins, minerals and fibres according to Kim et al. [2] and Amin and Nagy [3]. Calculations of HFD was 20% crude protein, 35% fat, 40% CHO (starch 35%, 5% sucrose) 5% vitamins and minerals and fibres. Metabolic energy of this diet is 5130 Kcal/kg 61% of this energy from fat.

**c- High sucrose diet**, consists of 600 gm sucrose, 250 gm concentrate, 100 gm methionine, 50 gm fibers, vitamins and minerals. The chemical analysis of this diet is 20% crude protein, 5% fat, 5% starch, sucrose 65% and 5% fibres, vitamins and minerals according to D'Alessandro et al. [4]. Metabolic energy of this diet is 3410 Kcal/kg. 73% from this energy produced from sucrose.

#### 2- Experimental animals

The present study carried out on male Wister rat range from 100-130 gm body weight, 8 wks old. They were purchased from the, Hellwan, Cairo, Egypt.

Rats were kept under observation for one wk before onset of experiment to be acclimatized and housed individually in metal cages at room temperature 37°C, less than 12 hrs light dark cycle in the laboratory of biochemistry in Faculty of Veterinary Medicine-Beni Suef University during the period of the research. Water and special diet were allowed to rats in free manner. Body weights were recorded weekly and food consumption was calculated daily.

#### 3-Drugs for treatment

*Garcinia*, drug used by dissolving one capsule in 10 ml D.W each capsule containing 500 mg hydroxy citric acid (HCA) were purchased from the EVA Pharm.

Oral administration of a dose of 50 mg/rat daily was done according to the method described by Ohia et al. [5]. Handling of the animal was the same for all groups and did not affect weight gain. The drug were given to rat by industrialized stomach tube and given slowly to rat stomach.

## Methods

### 1-Experimental design and animal grouping

Our experiment continued for 12 wk and divided into two phases (induction period and treatment period) and the whole number of rats was 32.

*a- Induction obesity period*: It began from 1st-8^th ^wk; by feeding rats with HFD or HSD. The animals were divided into three groups; control, HFD and HSD group.

-*Control group*, maintained on standard normal rat chow diet along the all period of experiment (12 wk) (8 rats).

*- HFD *group was maintained on high fat diet (8 rats). HSD was giving high sucrose diet (16 rats).

*b- Treatment period*: It began form 8-12^th ^wk during this period each of HFD and HSD group was divided into two groups, (HFD) group continued on fat diet and (HFD+G) treated group maintained on high fat diet to 4 wks with administration of *Garcinia *treatment at dose (50 mg/rat/day) by stomach tube. While sucrose group divided into HSD maintained on high sucrose diet (8 rats) and (HSD+G) maintained on high sucrose diet with *Garcinia *treatment (8 rats).

*Calculation of average food consumption *per each rat was recorded daily by subtracting the amount of food remaining in each day from the measured amount of food provided at the previous day [6].

*Calculation of energy intake of consumed diet: *Metabolic energy of standard rat chow, HFD and HSD were 2813, 5130 and 3410 Kcal/kg respectively. The average energy intake of consumed diet was calculated by multiplying the average consumed diet by 2.813, 5.130 and 3.410 respectively according to consumed diet [6].

BMI for rats was measured every wk and calculated by dividing the body weight in gm by the length (nose to base of tail) in cm^2^. Also body weight was measured.

### 2- Sampling and tissue preparation

Blood samples were collected from medial canthus of the eye, via glass capillaries at fasting state. The samples were collected in dry glass centrifuge tubes, allowed to coagulate at room temperature and centrifuged at 3500 rpm for 15 minutes at room temperature for separation of serum. The clear, non-haemolysed supernatant sera were separated using clean dry disposable plastic syringes and stored at -20°C for subsequent biochemical measurements. Besides addition of sodium fluoride as anticoagulant for measuring of glucose in another blood sample.

**Tissue sampling: **At the end of experiment, rats were sacrificed by decapitation and abdominal incision was immediately done after taking of blood sample for separation of kidney and perirenal, mesentric and epididymal adipose tissues. Kidney was taken and washed by saline, dried by filter paper and weighed 0.5 gm of this tissue then underwent homogenization for tissue biochemical analysis of catalase, MDA and NO.

### 3- Biochemical assays of serum and tissue

Plasma glucose was estimated according to the method of Trinder [7] using Stanbio Laboratory USA Kits. Serum urea [8] and creatinine levels [9] were measured colorimetrically using kits purchased from Diamond Diagnostic, Egypt. Serum was analyzed for lipid profile (TC, TG, LDL and HDL) levels and G6PD activity [10] by enzymatic colorimetric methods using assay kits (Bio-diagnostic Dokki, Giza, Egypt).

Renal and serum MDA levels were measured, as described by Mihara and Uchiyama [11]. Briefly, MDA was allowed to react with thiobarbituric acid that yielded red-coloured products, which were spectrophotometrically quantified by measuring the maximum absorption peaks at 532 nm. Renal catalase (CAT) activity was determined according to the procedure of Cohen et al. [12]. Renal and/or serum NO levels were measured according to Miranda et al. [13].

### Statistical analysis

Data were presented as mean ± S.E.M and analyzed using one-way analysis of variance (ANOVA) followed by Tukey-Kramer methods for post-hoc analysis. A value of p < 0.05 was considered statistically significant. Correlation coefficient between the measured variables was tested where (P) and (r) values were recorded. Graph Pad Prism 5 software (San Diego, CA, USA) was used for statistical analysis.

## Results

### Effect of HFD, HSD and *Garcinia *on feed intake, BW and BMI

**Figure 1:**
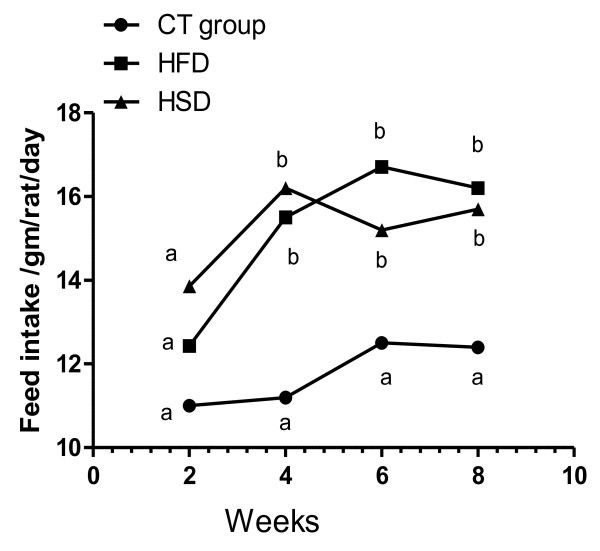
**Means of feed intake (gm/day/rat) in the different groups of experiment (0 to 8^th ^wk) in rats**. Feed intake increased significantly in rats fed on HFD and HSD from 0 to 8^th ^wk compared with that of control group. Means have different letters indicate significant variation at (P ≤ 0.05), while the same letters indicate non significant variation.

**Figure 2:**
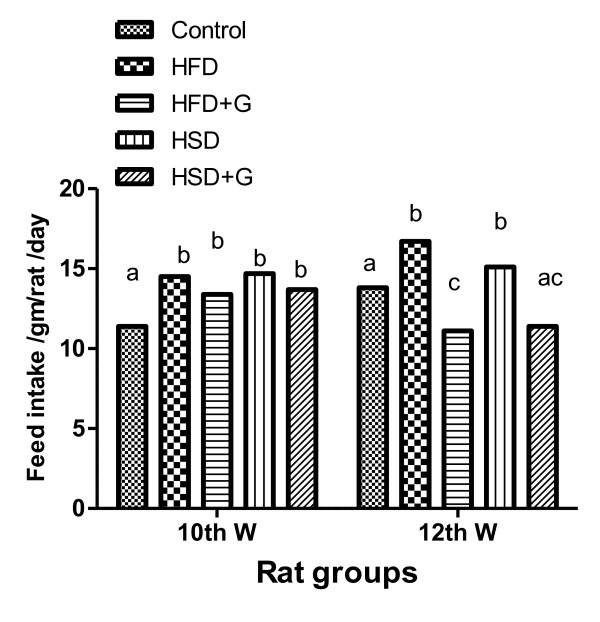
**Means of feed intake (gm/day/rat) in different groups of experiment (8^th ^-12^th ^wk) in rats**. Feed intake increased significantly in rats fed on HFD and HSD from 8^th ^-12^th ^wk compared with control group. *Garcinia *treatment tended to decrease feed intake compared with HFD and HSD group. Means have different letters indicate significant variation at (P ≤ 0.05)

**Figure 3:**
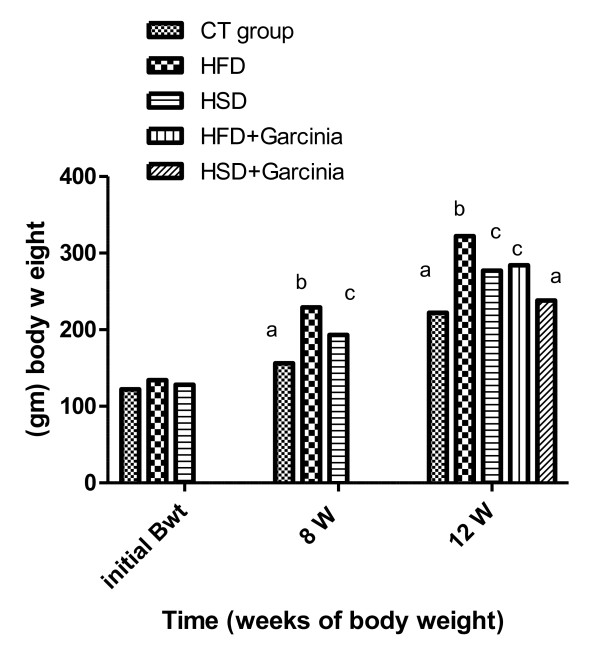
**Means of BW in rats during all period of experiment (Three months)**. Mean BW significantly increased during the period of the experiment (8^th ^-12^th ^wk) in HFD and HSD compared with that of control group. While *Garcinia *treatment significantly ameliorated the changes during 8^th ^-12^th ^wk compared with HFD and HSD group. Means have different letters are significantly different (p < 0.05).

**Figure 4:**
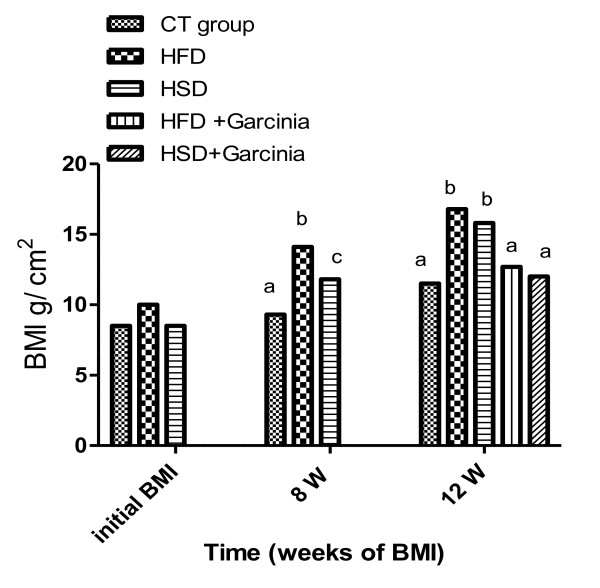
**Means of BMI in rats during the period of the experiment (Three months)**. BMI significantly increased during the period of the experiment in HFD and HSD compared with that of control group in rats. Administration of *Garcinia *significantly improved BMI compared with HFD and HSD group. Means not sharing common letter are significantly different (p < 0.05).

In this study, feed intake (Figure 1&2), BW (Figure 3) and BMI (Figure 4) were significantly increased during the period of the experiment in accompanied with significant increases in mesenteric, perirenal and epididymal adipose tissue in HFD and HSD compared with that of control group in rats. *Garcinia *treatment tended to decrease food intake, BW and BMI (Figure 2, 3, 4).

### Effect of HFD, HSD and *Garcinia *treatments on lipid profile and kidney function

Serum urea and creatinine levels were significantly increased in HFD and HSD compared with those in control group. There were significant higher levels of urea and creatinine in HFD than HSD group p ≤ 0.05. In addition, TC, triglyceride and LDL levels were also significantly increased in group HFD and HSD compared with those in control group. Cotreatment of HFD and HSD-administered rats with *Garcinia *produce significant decrease in serum urea, creatinine, TC, triglyceride and LDL levels compared to their respective HFD and HSD-treated rats (Table 1).

**Table 1:** Effect of HFD, HSD and *Garcinia *treatments on kidney function and lipid profile in different groups of rats.

	Control	HFD	HFD+ G	HSD	HSD+ G
**Creatinine (mg/dl)**	0.3 ± 0.02^a^	0.8 ± 0.03^b^	0.4 ± 0.04^ac^	0.8 ± 0.04^b^	0.3 ± 0.04^ac^

**Urea (mg/dl)**	19 ± 1.5 ^a^	38 ± 2.4 ^b^	14 ± 0.91^ac^	30 ± 3.9 ^d^	15 ± 1.8 ^ac^

**TC (mg/dl)**	81 ± 1.4^a^	351.01 ± 4.6^b^	112 ± 1.3^c^	320 ± 3.3^b^	172 ± 4.5^e^

**TG (mg/dl)**	85 ± 2.3^a^	384.01 ± 11^b^	203 ± 2.7^c^	350 ± 3.9^bd^	201 ± 10^ce^

**LDL (mg/dl)**	26 ± 2.1^a^	284 ± 5.5^b^	61 ± 3^c^	212 ± 4.9^d^	61 ± 3.9^c^

**HDL (mg/dl)**	44 ± 1.8^a^	73 ± 2.2^a^	72 ± 2.8^a^	77 ± 6.5^a^	66 ± 2.3^a^

Each value is the mean ± SEM. Means have different superscript letters indicate significant variation at (P ≤ 0.05), while the same letters indicate non significant variation

Serum urea and creatinine levels were significantly increased in HFD and HSD compared with control group. In addition, TC, triglyceride and LDL levels were also significantly increased in group HFD and HSD compared with those in control group. HFD and HSD-administered rats with *Garcinia *produced significant decrease in serum urea, creatinine, TC, triglyceride and LDL levels compared to their respective HFD and HSD-treated rats.

Effect of HFD, HSD and *Garcinia *treatments on blood glucose, G6pD and adipose tissues during three months in different groups of rats

### Effect of HFD, HSD and *Garcinia *treatments on plasma glucose, G6PD and adipose tissues

Table (2) showed significant increase in glucose in HFD group compared with control and these levels were significantly lowered in HFD+G than HFD group p ≤ 0.05. G6PD activities were significantly decreased in HFD while there were significant higher G6pD activities in HSD and HSD+G groups p ≤ 0.05 than control.

There were significant increases in mesenteric, perirenal and epididymal adipose tissues in HFD and HSD compared with that of control group in rats. *Garcinia *treatment tended to decrease adipose tissues weight compared with HFD and HSD group (Table 2).

**Table 2:** Effect of HFD, HSD and *Garcinia* treatments on blood glucose, G6pD and adipose tissues during three months in different groups of rats

	Control	HFD	HFD+G	HSD	HSD+G
**Glucose (mg/dl)**	59.0 ± 1.6 ^a^	111.0 ± 4.1^b^	91.0 ± 3.9^c^	71.0 ± 3.2 ^ad^	63.0 ± 3.6 ^ad^

**G6pD (mU/ml)**	0.25 ± 0.02^a^	0.14 ± 0.01^b^	0.12 ± 0.002^b^	0.53 ± 0.04^c^	0.54 ± 0.03 ^c^

**Mesenteric fat (g)**	1.7 ± 0.19^a^	3.6 ± 0.41^b^	2.6 ± 0.29^c^	3. 2 ± 0.29^c^	2.2 ± 0.33 ^e^

**Perirenal fat (g)**	2.4 ± 0.29^a^	3.3 ± 0.23^b^	1.3 ± 0.31^ac^	3.4 ± 0.2^bc^	2.2 ± 0.3^d^

**Epididymal fat (g)**	0.88 ± 0.06^a ^	2.3 ± 0.075^b^	1.1 ± 0.041^c^	1.9 ± 0.025^b^	*1.2 ± 0.1^dc^*

Each value is the mean ± SEM. Means have different superscript letters indicate significant variation at (P ≤ 0.05), while the same letters indicate non significant variation

Plasma glucose was significant increased in HFD group compared with control and these levels were significantly lowered in HFD+G than HFD group p ≤ 0.05. There were significant increases in G6pD activities in HSD and HSD+G groups p ≤ 0.05 than control, while giving *Garcinia *ameliorated these changes.

Mesenteric, perirenal and epididymal adipose tissue weight were significantly increased in HFD and HSD compared with control group.

The mesenteric, perirenal and epididymal fat-pad weights were reduced by 28%, 61% and 52% respectively in rats fed HFD+G than HFD rat (p < 0.05). While the mesenteric, perirenal and epididymal fat-pad weights were reduced by 31%, 35% and 37% respectively, in rats fed HSD+G than HSD rat. *Garcinia *treatment tended to decrease adipose tissues weight compared with HFD and HSD

Effect of HFD, HSD and *Garcinia *treatments on renal catalase and both serum and renal MDA and NO levels in rats.

The mesenteric, perirenal and epididymal fat-pad weights were reduced by 28%, 61% and 52% respectively in rats fed HFD+G than HFD rat (p < 0.05). While the mesenteric, perirenal and epididymal fat-pad weights were reduced by 31%, 35% and 37% respectively in rats fed HSD+G than HSD rat (p < 0.05) (Table 2).

### Effects of diet induced obesity & treatment on oxidative stress (MDA) and catalase activity

Feeding of HFD and HSD caused a significant increase in serum MDA level compared with control group. Treatment with *Garcinia *significantly decreased MDA level compared with those in group HFD and HSD. In renal tissues MDA level and catalase activity were significantly increased in HFD and HSD compared with control group and treatment with *Garcinia *ameliorated theses changes (Table 3).

**Table 3:** Effect of HFD, HSD and *Garcinia* treatments on renal catalase and both  serum and renal MDA and NO levels in rats

Parameters	Control	HFD	HFD+G	HSD	HSD+G
**Renal Catalase (U/gm)**	4.4 ± 0.29^a^	14.2 ± 1.3^b^	8.3 ± 0.64^c^	14.5 ± 0.34^b^	9.8 ± 0.67^c^

**S. MDA (nmol/ml)**	2.6 ± 0.22 ^a^	4.9 ± 0.31^b^	2.4 ± 0.42^a^	7.3 ± 0.37^c^	4.1 ± 0.44^b^

**Renal MDA (nmol/g)**	1.2 ± 0.15^a^	6.6 ± 0.27^b^	3.7 ± 0.41^cd^	4.8. ± 0.5^c^	2.7 ± 0.30 ^ad^

**S. NO (μmol/L)**	2.8 ± 0.28 ^a^	2.2 ± 0.14^b^	3.7 ± 0.24^ac^	1.6 ± 0.26 ^d^	6.8 ± 0.53 ^e^

**Renal NO (μmol/g)**	1.3 ± 0.09^a^	0.7 ± 0.04^b^	0.9 ± 0.025^c^	0.6 ± 0.03^b^	0.8 ± 0.02^c^

Each value is the mean ± SEM. Means have different superscript letters indicate significant variation at (P ≤ 0.05), while the same letters indicate non significant variation

Feeding of HFD and HSD caused a significant increase in serum MDA level compared with control group. Treatment with *Garcinia *significantly decreased MDA level compared with those in group HFD and HSD. In renal tissues MDA level and catalase activity were significantly increased in HFD and HSD compared with control group and treatment with *Garcinia *ameliorated theses changes.

Both serum and renal NO levels significantly lowered in HFD and HSD in comparison with control. Treatment with the *Garcinia *significantly elevated the No level in rats consuming HFD and HSD.

### Effect of diet induced obesity & treatment on serum and renal NO levels

HFD and HSD significantly lowered both serum and renal NO levels in comparison with control. Treatment with the *Garcinia *significantly elevated the No level of the rat consuming HFD and HSD (Table 3).

### Correlation coeffecient between variables in different groups of rats

There were significant and positive correlation between increased level of LDL and oxidative stress which was demonstrated by high level of MDA (P = 0.02 & r = 0.94) (Table 4). Also were significant positive correlations between TG and Oxidative renal MDA marker (P = 0.01 & r = 0.96) (Table 4).

**Table 4:** Correlation coeffecient between variables in different groups of rats.

	TG	LDL	Creatinine	BMI	BW	Renal Catalase	Renal NO
**TG**	____	P = 0.01 r = 0.95	P = 0.02 r = 0.93	P = 0.01 r = 0.96	P = 0.03 r = 0.92	P = 0.003 r = 0.98	P = 0.02 r = -0.94

**Creatinine**	P = 0.02 r = 0.93	P = 0.01 r = 0.96	__	P = 0.002 r = 0.98	P = 0.04 r = 0.89	P = 0.04 r = 0.89	NS

**Urea**	P = 0.04 r = 0.88	P = 0.002 r = 0.98	P = 0.02 r = 0.94	P = 0.006 r = 0.97	NS	NS	NS

**Renal MDA**	P = 0.01 r = 0.96	P = 0.02 r = 0.94	P = 0.042 r = 0.89	P = 0.02 r = 0.94	P = 0.01 r = 0.97	P = 0.04 r = 0.90	P = 0.046 r = -0.88

**Renal Catalase**	P = 0.003 r = 0.98	P = 0.03 r = 0.90	P = 0.04 r = 0.89	P = 0.03 r = 0.91	NS	____	P = 0.005 r = -0.97

**Renal NO**	P = 0.04 r = -0.90	NS	NS	NS	NS	P = 0.005 r = -0.97	____

**BMI**	P = 0.01 r = 0.96	P = 0.001 r = 0.9	P = 0.002 r = 0.98	____	P = 0.03 r = 0.92	P = 0.03 r = 0.91	NS

*r = Value of correlation coefficient P ≤ 0.05 indicate significant variation*.

There were significant and positive correlation between increased level of LDL and oxidative stress which was demonstrated by high level of MDA (P = 0.02 & r = 0.94) (Table 4). Also were significant positive correlations between TG and Oxidative renal MDA marker (P = 0.01 & r = 0.96) (Table 4).

The current data showed negative correlation between renal NO with renal MDA (P = 0.046 and r = -0.88) and renal catalase (P = 0.005 and r = -0.97) as indicator for oxidative stress in kidney. Moreover there were significant positive correlations among BMI, kidney functions as creatinine (P = 0.98 &r = 0.002) and urea (P = 0.006 & r = 0.97).

The current data showed negative correlation between renal NO with renal MDA (P = 0.046 and r = -0.88) and renal catalase (P = 0.005 and r = -0.97) as indicator for oxidative stress in kidney. Moreover there were significant positive correlations among BMI, kidney functions as creatinine, (P = 0.98 &r = 0.002), urea (P = 0.006 & r = 0.97).

## Discussion

### Effect of diet induced obesity and treatment on feed intake, body weight and BMI

In this study there was significant higher food intake in both free diet of HFD and HSD during 4^th^, 6^th ^and 8^th ^wk (Figure 1) and 10^th ^&12^th ^wk (Figure 2) compared to control diet and this is may be due to HFD causing hyperphagia which similar to human cafeteria diet [14]. The mechanisms for how saturated fat and sugar-based beverages contribute to human obesity are clearly in rats on an HF choice diet, plasma leptin concentrations and proopiomelanocortin mRNA increased and neuropeptide Y mRNA decreased [15].

Dietary fat possesses a number of characteristics that may contribute to its overconsumption. Both palatability and energy density were contribute to fat hyperphagia and reduced satiation signaling accompanying HFD consumption which could contribute to overconsumption and often leads to obesity. Although HF adaptation promotes short-term overconsumption of a high-energy food (i.e., reduced satiation), it also appears to offer a vital influence in the control of energy metabolism. Long-term HFD eating may promote excessive short-term energy intake by reducing sensitivity to the satiating properties and thus consume more food [16].

Rats fed high-saturated fat had both hyperglycaemia and hyper-triacylglycerolemia, the same as our model while rats fed high n-3 PUFA only had hyperglycaemia. In this concern various forms of dietary fat differentially change the expression of neuropeptide genes involved in energy homeostasis [17].

Higher feed intake in HSD may be due to hyperphagia induced by sucrose which affect appetite centers in hypothalamic neuropeptids Y, (NPY) and proopinne melanocortin (POMC) both receive information about nutritional status and level of energy storage through insulin and leptin signaling mediated by specific receptor located POMC and NPY neurons. Sucrose consumption increase calories intake through up regulation of hypothalamic CB1 mRNA and down regulation of NPY mRNA [17]. Also consumption of sucrose solution results in high body weight gain through activation of hunger signals and depression of satiety signals which support our results.

The current data showed significant increase in body weight and BMI especially in the 8^th ^wk in HFD and HSD (Figure 3 &4). Giving of HFD through canula showed significant increase in body weight in accordance with Akiyama et al. [18].

Increased BW and BMI may be due to increase caloric intake resulting in more adipose tissue deposition than starch diet this result varies between researches due to sex, age, strain. Furthermore HSD appear to induce mitochondrial dysfunction in adipose tissue, which may be related to greater weight gain and metabolic impairment [19,20].

*Garcinia *ameliorated feed intake (Figure 2) and significantly decreased BW and BMI (Figure 3&4). These effects of HCA in *Garcinia *achieved by increasing release/availability of 5 hydroxy tryptamine, or serotonin, also enhance serotonin uptake in the brain [5]. Serotonin, a neurotransmitter implicated in the regulation of eating behavior, appetite control and weight management by curbing appetite, reduction of food intake and inhibiting body fat biosynthesis [21].

### Effect of diet induced obesity and treatment on lipid profiles

HFD and HSD significantly increased TC, TG and LDL levels in comparison with control group (Table 1). These findings may be owed to high fat from beef tallow induced hypercholesterolemia [22]. Dietary sucrose significantly produced hypertriglyceridemia across rats' life span that had free food access or were calorie restricted and may be due to increased secretion of TG and decreased its catabolism [23]. HSD induces hepatic synthesis of TG from glucose and transport it to blood through VLDL and stored in adipose tissue.

There were significant and positive correlation between increased level of LDL and oxidative stress which was demonstrated by high level of MDA (P = 0.02 r = 0.94) (Table 4) and high MDA from lipid peroxidation causing oxidative stress which generally result in increase oxidation of LDL.

*Garcinia *treatment produces decrement in LDL, TG and TC serum levels. These findings occur as a result of inhibiting effect of hydroxy citric acid on ATP citrate lyase, an enzyme which catalyses extramitochondrial cleavage of citrate to oxaloacetate and acetyl COA. The produced acetyl COA is used in fatty acid synthesis, TC and TG synthesis. The current data suggested that *Garcinia *has hypolipidemic action causing significant hypocholesterolemic and hypotriglycerdemic effect.

### Effect of HFD, HSD and treatment on kidney function

Lifestyle factors and diet play a role in the development of kidney disease in several stages including, progress of obesity and the metabolic syndrome and occurrence of obesity-related glomerulopathy.

HFD and HSD caused significant elevation in serum creatinine a specific indicator of glomerular function and urea level (Table 1) owing to consumption of HFD and HSD which result in metabolic syndrome marked by obesity, hyperlipidemia and associated with oxidative stress and nitric oxide inactivation by reactive oxygen species (ROS) and diminish NO bioavailability [24] which leading to renal dysfunction, characterizing by high level of creatinine and blood urea nitrogen [25].

Using HFD (32%) for 10 wk, about one-half develop obesity and mild hypertension, oxidative stress and impaired renal function. Also hydroxynonenal protein adducts in the kidney were highly increased, indicating oxidative stress in the renal tissue [26].

In obese subject, increased serum creatinine was to be observed suggesting that obesity caused elevation in renal function test and produce proteinuria, concomitantly with other risk factors such as hypertension, diabetes and dyslipidemia [27].

Obesity is recently acknowledged as an important independent risk factor for kidney disease. This risk is probably explained by renal intracellular lipid accumulation [28]. Also metabolic disorders in obesity associated with high blood pressure, poor glycemic control, dyslipidemia and smoking were considering risk factor for susceptibility of chronic renal disease (CRD). Glomerular hypertension and endothelial dysfunction were regarded as common mechanism of CRD rennin-angiotensin system.

Renal triglyceride accumulated in obese rats accompanied by hypoalbuminemia and elevated blood urea nitrogen. Dysregulated gene expression may result in increased renal collagen cross-linking and lipid accumulation, that may be associated with development of nephropathy in the animal model of type 2 diabetes and obesity [29].

Feeding HFD to rat resulted in macroscopical and microscopical changes in kidney weight, total kidney volume, changes in medulla and cortex, glomerulus, proximal and distal convoluted tubules [30].

Visceral and renal fat accumulation through consumption of a HFD leads to marked renal sympathetic activation, which is related to increased responsiveness to central sympathoexcitatory effects of leptin that contributes to the development of hypertension [31].

Insulin resistance and increased sympathetic nerve activity were implicated in development of glomerular hypertension and endothelial dysfunction [32]. Furthermore hyperinsuliemia induced by obesity cause relaxation of afferent arterioles leading to hyperfiltration of gloumerulus and renal damage [33], which explained the defect in renal functions in our model of HFD and HSD.

*Garcinia *treatment enhances renal function as a result of HCA-SX derived from Garcinia cambogia (HCA-SX, Super CitriMax) which attenuated the increased oxidative stress biomarker through reducing lipid peroxidation (MDA) and declining lipid profiles and level of oxidized LDL which generally improved kidney function [34].

### Effect of diet induced obesity and treatment on blood glucose level

Obesity and type 2 diabetes mellitus is correlated with each other, in our experiment significant hyperglycaemia occurred due to HFD (Table 2), which resulted from free fatty acid release from adipose tissue causing peripheral and hepatic insulin resistance initiating T2DM. Furthermore, FFA impair muscle uptake of glucose by competitive inhibition [[18] &[35]].

HSD has no significant effect on blood glucose level, that result was in accordance with Fukuchis et al. [35] who reported that rats fed a sucrose diet for 4 wks had significantly larger visceral fat pads and hypertriglyceridemia, however, neither plasma glucose nor insulin levels were significantly higher, while hyperglycemia and insulin resistance occur after 20 wks of feeding HSD.

Relationship between renal disorder and obesity in HFD group was confirmed by hyperglycemia, insulin resistance, decreased production of renal NO and increased oxidative stress which implicated in progression of diabetic nephropathy [36].

*Garcinia *significantly improved the hyperglycemia, in HFD, by declining insulin resistance [37], in this concern *Garcinia *could be used for management of diabetes, by increasing metabolic pathway via rising glucose oxidation through improving insulin action; also Garcinia promotes glycogenesis and lipid oxidation.

### Effect of diet induced obesity and treatment on G6PD activity

G6PD activities were significantly decreased in HFD (Table 2). These findings may be due to hyperglycemia with HFD caused activation of protein kinase A and subsequent increased phosphorylation and inhibition of G6PD activity and hence decreased NADPH which therefore led to increased oxidative stress [38]. G6PD activity and accordingly NADPH level was significantly decreased in diabetic nephropathy.

Our data show that hyperglycaemia during HFD can reduce G6PD activity. Conversely, G6PD insufficiency can promote oxidative stress and impairment of insulin secretion by beta cells. It is suggested that reduced G6PD activity and HFD can aggravate each other, and HFD could be aetiologically associated with reduced G6PD activity.

Lipid peroxidation was significantly increased, while G6PD activity was decreased in HFD group. Oxidative stress due to increased oxidant production and/or decreased antioxidant activity is a critical underlying mechanism. The principal intracellular reductant is NADPH whose production is mainly dependent on G6PD activity.

There was significant increase in G6pD activities in HSD and HSD+G groups p ≤ 0.05 compared with control (Table 2) which may be attributed to metabolic alteration produced from sucrose rich diet, among these changes increased level of G6PD which is the first enzyme of hexose monophsphate pathway and induction of G6pD in paranchymal cells that support the escalating needs for NADPH for synthesis of fatty acids [39].

### Effect of diet induced obesity and treatment on adipose tissue

Our data indicated significant increases in mesenteric, perirenal and epididymal adipose tissues in HFD and HSD group (Table 2). HFD increases the weight of different fat depots resulting in significant decrease in OB-Rb mRNA level [40].

Adipose tissue functions as an endocrine organ, secreting hormones/cytokines (e.g., leptin) which may trigger renal sodium retention by activating the renin-angiotensin. Furthermore, excess perirenal and visceral adipose tissue may physically compress the kidneys, increasing intrarenal pressures and tubular reabsorption. Ultimately, continuous obesity causes renal structural alteration, glomerular hypertrophy and occasionally focal segmental glomerulosclerosis [41].

HCA, an active ingredient of *Garcinia*, reduces food consumption probably by diverting fatty acids and carbohydrates that would have become fat in the liver into hepatic glycogen [5]. This metabolic alteration may send a signal to the brain that result in rising serotonin level concomitant with a reduced appetite. Moreover, using *Garcinia *causes dispersion of fat which facilitate action of lipase on adipose tissue [42], suppresses body fat accumulation [43], inhibit cytoplasmic lipid accumulation and regulates adipogenesis, so eliminates body fat.

### Effect of diet & treatment on renal catalase activity

Renal catalase activity was significantly increased in HFD and HSD (Table 3). These findings occur in response to oxidative stress and important to balance the elevated ROS resulting from the activation of biochemical pathway leading to increased level of ROS and increased lipid peroxidation, producing oxidative stress [35] . Increased catalase activity was indicative of elevated ROS and consequently higher oxidative stress.

The increment in renal catalase and MDA offer better understanding and evidence for the relation between obesity and oxidative stress where increased ROS levels generally stimulate antioxidant system as a compensated defense mechanism and are an important trigger for insulin resistance. Some researchers reported that when exposure of alpha cells of pancreas to diabetogenic substance there was increase of catalase activety [44].

Renal catalase activity reduced after using *Garcinia*, suggesting the role of HCA-SX to attenuate the increase in oxidative stress, via decreasing feed intake, indicating the antioxidant effect of *Garcinia*.

### Effect of diet induced obesity and treatment on serum and renal MDA

HFD and HSD generated significant increase in blood and renal levels of MDA compared to control group, while *Garcinia*, significantly decreased levels of serum MDA and renal MDA compared with HFD and HSD group(Table 3) as result of HCA-SX that inhibit ATP citrate lyase which catalyse extramitochondrial cleavage of citrate to oxaloacetate giving acetyl COA which used in fatty acid synthesis, suggesting that *Garcinia *has hypolipidemic action and reduces MDA in kidney, so HCA improves lipid peroxidation [34].

Feeding HFD and HSD raised LDL, TC, TG and renal lipid peroxidation (MDA) level which affect on the kidney because MDA act as tissue toxicant metabolites. These changes were monitored by increased level of urea and creatinine, whereas using *Garcinia *as antiobesity agent decreased TC, LDL and improved kidney function.

A number of reports suggested that ROS overproduction in the kidneys [36], heart and arteries [45], was involved in obesity-induced hypertension. However, the role of ROS and NO in the kidney has not been clarified.

The current data showed that TG positively correlated significantly with renal MDA (P = 0.01 and r = 0.96) and catalase (P = 0.003 and r = 0.98) as indicators of oxidative stress, while negatively correlated with renal NO, (P = 0.04 and r = -0.90) (Table 4) suggesting an association between renal lipid peroxidation and obesity associated nephropathy. Furthermore, oxidative stress was increased in HFD and HSD groups as MDA level and catalase activity were increased, while NO level was decreased.

### Effect of diet induced obesity & treatment on serum and renal NO level

HFD and HSD significantly decreased serum and renal NO compared with control group (Table 3). Obesity associated with hyperglycemia is a key factor that contributes to the development of diabetes-related microvascular disease both cyclooxygenase1 and cyclooxygenase2 and play a key role in the regulation of cardiovascular function, this increment resulted in rise of oxidative stress and reduction in NO generation in microvessels endothelial cells [46], suggesting that NO deficiency may contribute to renal vascular congestion and the renal dysfunction progression.

NO synthetase activity in the HFD group was decreased associated with diminished L-arginine transportation [47]. Also adipose tissue has a role in secreting factor that impairs endothelial dependent dilatation via inhibition of NO synthase mediating NO production [48] . Consequently, *Garcinia *that decreased TG results in replenish of NO level to normal.

NO signalling, which is involved in the regulation of food intake and insulin signalling, is altered in obesity and diabetes. It was suggested that hyperglycaemia impairs glucose and insulin regulation of NO production which occur through AMP-activated protein kinase [49].

Our data showed negative correlation between renal NO with renal MDA (P = 0.046 and r = -0.88) and renal catalase (P = 0.005 and r = -0.97) as indicator for oxidative stress in kidney tissues (Table 4). These are considering novel data of our known, suggesting that renal oxidative stress associated with lower renal NO initiating glomerular vasoconstriction and favour nephropathy.

We found that NO negatively correlated with MDA and hence its inactivation by ROS and functional NO deficiency in our model. So enhanced ROS-mediated inactivation and sequestration of NO which may contribute to the reduction of bioactive NO in obesity and hypertriglyceridemia.

NO plays an important role in the regulation of renal blood flow to the renal medulla and in the tubular regulation of sodium excretion. Rats fed HFD, resulted in decrease in serum and renal NO production (Table 3), indicating that obese rats are more liable to develop hypertension. Furthermore, HFD -induced defects in NO production may promote the salt-sensitivity of blood pressure, which appears to require more NO to maintain blood pressure during a salt challenge [50].

NO decreased in HFD and HSD, initiate vasoconstriction that affect the kidney and using methods for controlling obesity generate increased levels of NO and improving state of the involved organs that apparent in our results due to action of NO as vasodilator via using *Garcinia*.

Physical activity as a method of management of obese person prevent cardiovascular disease by increasing NO production and lessening NO inactivation [51], which could be comparable with our model of *Garcinia *used as antiobesity drugs.

Our rat's model was developed obesity and hyperglycemia by 12 wks on HFD and HSD diet. They also developed hypercholesterolemia, profound hyper-triglyceridemia, high urea and creatinine, and renal function disorders. Furthermore, serum NO production was decreased, and homogenates from kidney demonstrated reduced NO. Our data offer novel insights into potential mechanisms of renal dysfunction, oxidative stress and NO in obesity and demonstrate the efficacy of HCA-SX in renal protection and weight management.

Collectively, feeding HFD and HSD to rats resulted in significant elevating in BW, BMI, feed intake, adipose tissue, blood levels of glucose, TG, TC, LDL, MDA and catalase activity while significantly, decreased serum and renal NO levels in our model. All of these parameters implicated in renal disorders, consequently produce adverse effect on kidney, while administration of *Garcinia *improves these changes as, HCA-SX attenuates the increase in oxidative stress so improves lipid peroxidation which is cytotoxic by reducing free-radical production and by increasing nitric oxide production/availability.

## Conclusions

We could concluded that HFD and HSD induced obesity, represent the best available rat model to study nephropathy, which riskily affect the kidney via elevating urea, creatinine, TG, LDL and glucose and that the renal injuries were associated with increased oxidative stress monitored by increased MDA level and catalase activity whereas NO production decreases while using *garcinia *cambogia improved the levels of blood lipid profile, oxidative stress, biomarkers and glucose so improving kidney function. This study provides novel in vivo evidences of the protective effects of *Garcinia *against obesity-induced nephropathy.

## Competing interests

The authors declare that they have no competing interests.

## Authors' contributions

KA, HK and MA carried out experimental work; biochemical analysis, statistical analysis, interpretation and discussion of the results related to their part of the work. KA conceived, design and planning of the study, wrote the paper, drafting and revision of the manuscript. All authors read and approved the final manuscript.
